# Mitigating nipple areolar complex (NAC) ischemia following mastopexy augmentation using nitroglycerin application and methylprednisolone: a case report

**DOI:** 10.1080/23320885.2023.2285056

**Published:** 2023-11-27

**Authors:** Thor S. Stead, Matteo Laspro, Giovanni Ramirez-Arroyo, Latha Ganti, Amitabha Mitra

**Affiliations:** a Department of Plastic Surgery, The Warren Alpert Medical School of Brown University, Providence, RI, USA; b Hansjörg Wyss Department of Plastic Surgery, NYU Grossman School of Medicine, New York, NY, USA; c Department of Emergency Medicine, University of Central FL College of Medicine, Orlando, FL, USA; d Department of Plastic Surgery, UPMC Williamsport Medical Center, Williamsport, PA, USA

**Keywords:** Mastopexy, breast augmentation, body contouring, ischemia, emergency department, nipple necrosis

## Abstract

A patient who underwent bilateral wise-pattern mastopexy augmentation with threatened nipple-areolar complex necrosis was successfully managed medically with IV methylprednisolone and topical nitroglycerin ointment as immediate consultation with the operating surgeon was not possible.

## Introduction

Necrosis of the nipple-areolar complex (NAC) is a rare, but dreaded complication following nipple-sparing mastectomy, mastopexy, breast reduction and breast augmentation. The blood supply of the NAC arises from the intercostal and internal mammary arteries which are often not identified intraoperatively and damage to them can form a hematoma leading to impaired tissue perfusion and ischemia that results in skin necrosis. This may ultimately threaten implant exposure which requires removal and secondary procedures that compromise a satisfactory outcome [1]. Postoperative guidelines following breast augmentation are designed to mitigate the most common complications, such as infection or wound dehiscence. Yet, they often do not address ischemia. Furthermore, there is a scarcity of evidence-based directives to rectify impeding necrosis by an independent provider other than the primary surgeon. A pertinent need for these directives has arisen recently due to the increase in patients traveling for their operations and thus being managed in local emergency rooms if/when complications arise.

The authors present a case of a patient in the emergency department (ED) three days post implant-based breast augmentation and mastopexy who was suspected of threatened ischemia of the NAC without wound dehiscence. The patient was successfully managed medically with IV methylprednisolone and topical nitroglycerin ointment as immediate consultation with the operating surgeon was not possible.

## Case presentation

A thirty-year-old female with no past medical history presented to the ED after-hours due to left breast pain that had been getting worse. The patient had bilateral breast augmentation and mastopexy with saline implants three days prior in a private plastic surgery practice out of town. The office was 4 h away, and she was unable to reach the surgeon’s office for guidance. At the time of presentation, the patient reported shortness of breath and an inability to take in liquids, despite feeling dehydrated. She also reported thoracic back pain, left greater than right. She had been taking amoxicillin-clavulanate twice daily since the day of surgery. On the night of the surgery, she visited the ED due to urinary frequency and right flank pain and was diagnosed with a urinary tract infection for which she was started on cephalexin in addition to the amoxicillin-clavulanate. The patient’s surgical history is notable for only a gastric band. Patient denied a history of current or past smoking. During her emergency room visit, she denied any fevers, chills, chest pain, vomiting, diarrhea, abdominal pain, or headache. Vital signs were significant for tachycardia at 132 beats per minute. Her initial blood pressure was 153/79 mmHg, with respiratory rate 18 breaths per minute, temperature of 97.9 °F and oxygen saturation of 99%.

**Figure 1.**
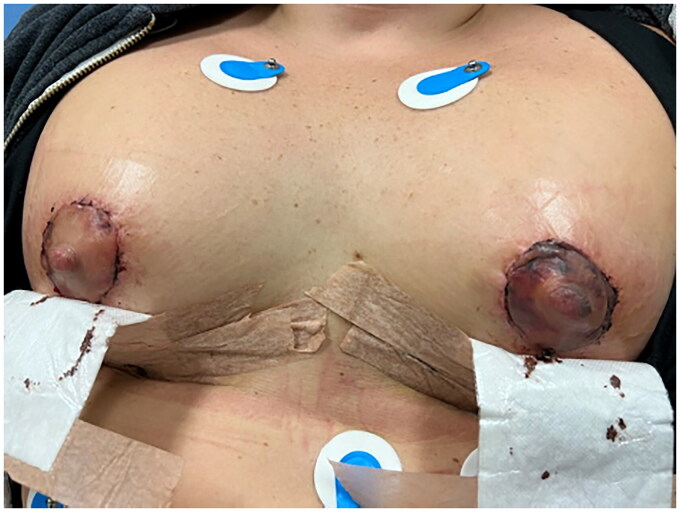
Photograph of patient’s breasts demonstrating periareolar incisions.

**Figure 2.**
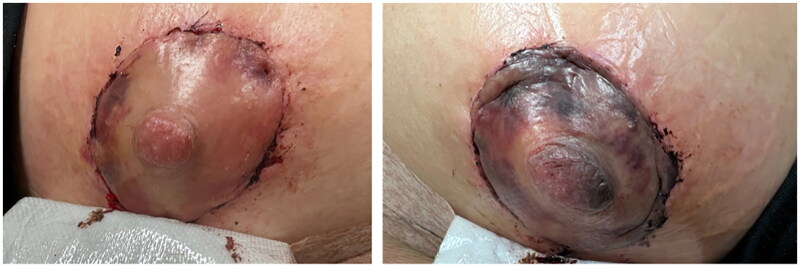
Photograph depicting significant discoloration of left NAC compared to right.

**Figure 3.**
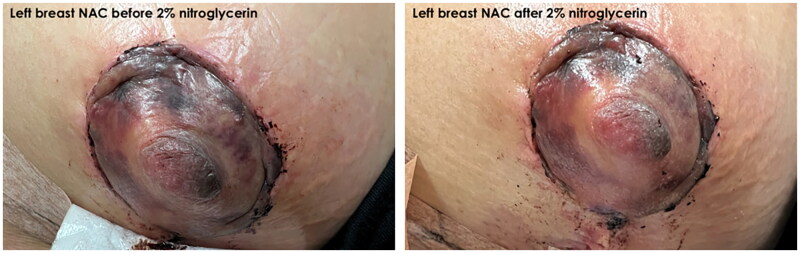
Comparison of NAC before (left) and after (right) application of 2% nitroglycerin ointment and intravenous methylprednisolone.

On physical examination, patient was found to have a Wise pattern mastopexy with a breast augmentation. There was mild erythema surrounding the areola of the left breast. There was no hematoma present, no drainage from the nipple, sutures were intact without evidence of wound dehiscence. Bilateral breasts were symmetric on inspection with no deformities and no fluid collection or infection. The inframammary incisions were found to be satisfactory without any erythema, drainage, or dehiscence. The overall breasts were swollen. The left NAC was found to be dusky, significantly darker and discolored compared to the right, indicative of venous congestion as well as impending necrosis (Figures 1 and 2). A small amount of 2% nitroglycerin ointment was applied to the NAC with some improvement in color within 30 min (Figure 3). It was also decided to administer intravenous methylprednisolone treatment to further alleviate the venous congestion and swelling, which may revert the chance of ultimate necrosis. As the primary surgeon was not available, removal of sutures was not attempted.

Top differential diagnoses for her tachycardia included dehydration, sepsis and pulmonary embolism. Sepsis workup was normal revealing only a mild leukocytosis of 11.3. She was given 3 L of isotonic saline over 6 h to address potential dehydration. Computed tomography angiography (CTA) of the chest ruled out pulmonary embolism. Since the patient was already on amoxicillin-clavulanate and cephalexin, she was given doxycycline for atypical bacterial coverage. She received intravenous morphine for analgesia.

Her discharge instructions were to follow up promptly with her surgeon first thing in the morning. Pictures of the areola, and all her lab and radiology results were given to the patient so she could send them to her surgeon. She received one more dose of 2% nitroglycerin for topical use in the morning and prescriptions for doxycycline and methylprednisolone. She was told to stop the cephalexin as it was redundant therapy.

Two days after her ED visit, she was happy to report that she was feeling better, that the discoloration of her nipple had resolved and that there was no partial or full thickness skin loss. This was a huge relief to her as she had been very worried about losing the nipple, a potential complication the surgeon had warned her about.

## Discussion

This case highlights the successful early management of ischemia to prevent NAC necrosis. The proposed mechanism of action for nitroglycerin in this setting is venodilation of patent vessels draining the NAC, which alleviates venous congestion and allows for greater tissue oxygenation. Additionally, arterial vasodilation of patent vessels supplying the NAC compensates for decreased blood flow from superficial vessels that may have been damaged intraoperatively or due to postoperative inflammation [2]. Topical nitroglycerin is rapidly acting, requiring only 5–10 min for full transdermal absorption, making it an ideal choice in acute care settings [3]. A retrospective study from 2017 has demonstrated the efficacy of low-dose topical nitroglycerin ointment in decreasing the rate of skin flap necrosis in skin/nipple sparing mastectomies [4]. This was later confirmed in a meta-analysis in 2020 [5]. In our case, reperfusion of the NAC was clearly visible 30 min after the first application. Adverse effects of the ointment are very limited and include flushing, rash and dermatitis, which resolve shortly after cessation of use [3]. Our patient did not exhibit any of these complications.

Alleviation of postoperative inflammation has also been shown to aid in NAC ischemia reversal. Methylprednisolone reduces local tissue swelling and promotes venous drainage of the NAC and serves as an easily administered, widely accessible medication that is appropriate following implant-based breast reconstruction [6]. A randomized controlled trial showed significantly lower rates of seroma formation in nipple-sparing mastectomy after local methylprednisolone injection [7]. The rationale for systemic administration in our case was due to the ongoing inflammation caused by the UTI our patient was recovering from, though evidence for systemic vs local methylprednisolone injection is scarce. Local injection of methylprednisolone is a viable alternative to systemic administration to prevent seroma formation and reduce the inflammation associated with ischemic tissue damage. Another RCT demonstrates that methylprednisolone may also have additional benefits after breast augmentation, such as reduced pain, fatigue and emesis [8]. Yet, especially in the case of large augmentations, infection rates are not negligible [1]. Considering the immunosuppressive role of methylprednisolone, proper weighing of risks and benefits before administration is required.

Following resolution of the ischemic state, gauging of nipple sensitivity following the initial vascular compromise is necessary. Nipple-areola sensibility evaluation has been explored in mammaplasty cases and has been shown to negatively correlate with the cutaneous pressure threshold [9]. While large-scale studies in mastopexy-augmentation have not yet been conducted, the authors postulate that measuring the cutaneous pressure threshold may be of use in determining nipple sensitivity following recovery from an ischemic insult. In the case of irreversible nipple necrosis, reconstruction may be necessary, and algorithms for determining the ideal method of reconstruction have been proposed [10].

The reversal of NAC ischemia through nitroglycerin ointment and methylprednisolone is an easily accessible intervention to extend flap viability for post-augmentation patients who cannot immediately see a surgeon due to physical constraints such as time or distance, cannot undergo debridement for any reason, or lack local resources to address the problem promptly. Both medications have extremely broad indications and are available at offices ranging from urgent care centers to freestanding EDs. Furthermore, these medications can be safely administered and followed by other physicians until the patient is able to be seen by a plastic surgeon, which dramatically increases patients’ accessibility to care and potential for NAC-saving intervention.

Nitroglycerin ointment use for NAC ischemia has been well-documented in nipple-sparing mastectomy (NSM), reduction mammaplasty and oncoplastic breast reconstruction, but reports of its efficacy in reversing NAC ischemia post-breast augmentation without mastectomy are scarce. The same is true of methylprednisolone, which has been studied in patients following breast augmentation, but not specifically for the purposes of reversing NAC ischemia. As periareolar incisions have been implicated in increased rates of NAC ischemia, addition of topical nitroglycerin in the post-operative regimen may be warranted even when immediate signs of necrosis are not present. Due to the low risk profile of the drug, prospective studies looking at nitroglycerin in the context of cosmetic and reconstructive breast surgery are needed to assess whether inclusion of the medication is desirable ubiquitously.

## Conclusion

This case proposes that concomitant use of nitroglycerin and methylprednisolone could successfully mitigate impending NAC ischemia without surgical intervention utilizing two nearly ubiquitously available medications in a freestanding ED.

## Consent form

Informed consent to publish the details of the case and the attached photos was obtained from the participant in this study.
